# Central, peripheral ECMO or CPB? Comparsion between circulatory support methods used during lung transplantation

**DOI:** 10.1186/s13019-021-01719-0

**Published:** 2021-11-27

**Authors:** Nikola Ruszel, Kajetan Kiełbowski, Maria Piotrowska, Michał Kubisa, Tomasz Grodzki, Janusz Wójcik, Bartosz Kubisa

**Affiliations:** Department of Thoracic Surgery and Transplantation, Independent Public Regional Hospital, Pomeranian Medical University, Sokołowskiego 11 Street, Szczecin-Zdunowo, Poland

**Keywords:** Lung transplantation, ECMO, CPB, ECC

## Abstract

**Background:**

Chronic obstructive pulmonary disease, cystic fibrosis and usual interstitial pneumonia are three most common indications for lung transplantation (LuTx) in Poland. As a result of irreversible destruction of pulmonary parenchyma and extended respiratory insufficiency that appear afterwards, it is crucial to estimate the reserve of gas exchange in each lung before and during surgery. Altering conditions of gas exchange require adaptation in circulatory system as well. In some of the cases the use of extracorporeal life support appears to be necessary to undergo the transplantation successfully. Cardiopulmonary bypass (CPB) or extracorporeal membrane oxygenation (ECMO) used during operation allow to replace the function of heart and lung, but they are also related to complications in the form of acute kidney failure, bleeding, heart arrhythmias or thromboembolic complications.

**Methods:**

We reviewed 77 LuTx from 2009 to 2020 performed at the Department of Thoracic Surgery and Transplantation. 40/77 (51%) patients required intraoperative extracorporeal assistance: 8 required CBP and 32 required ECMO. In the ECMO group 14/32 (44%) patients had peripheral cannulation and 18/32 (56%) had central one. We have calculated the survival rates and reviewed postoperative complications after lung transplantations. Cumulative Kaplan–Meier survival curves were calculated. Differences between the groups were evaluated by the Chi- square analysis for discontinuous variables and t-test for continuous variables.

**Results:**

The use of intraoperative central extracorporeal membrane oxygenator was associated with increased survival rates comparing to patients without external support (30-days, 1-year, 3-years, 5-years rates: 78%, 66%, 66%, 66% vs 83%, 65%, 59%, 44% respectively). Furthermore, survival was enhanced comparing to peripheral ECMO or cardiopulmonary bypass as well (50%, 41%, 41%, 33%; 75%, 50%, 50%, 38% respectively). Acute kidney injury and thromboembolic complications occurred statistically more often in case of patients that underwent lung transplantation with support devices (*p* = 0.005, *p* = 0.02 respectively). Frequency of other complications was comparable among groups.

**Conclusions:**

The use of central extracorporeal membrane oxygenation should be favorized over peripheral cannulation or cardiopulmonary bypass. CPB should be no longer used during LuTx.

*Trial registration* Not applicable.

## Introduction

Lung transplantation (LuTx) is a procedure used as an ultimate treatment of the irreversible destruction of pulmonary parenchyma and consequent respiratory insufficiency. It is dedicated to carefully selected group of patients, when pharmacological remedial solutions are no longer effective [1, 2]. However, the patient’s expected 2-year survival rate has to be lower than 50% and the patient is expected to have over 80% likelihood to survive the first 3 months after LuTx [1].

The first attempt of LuTx in Poland occurred in 1996 in Szczecin, when Grodzki carried out the living-donor lung transplantation (LDLT) and transplanted lower lung lobe to the patient suffering from usual interstitial pneumonia. Next year (1997) Zembala in Zabrze performed the single-lung transplantation (SLuTx). Soon this "treatment of last chance" began to develop effectively in Poland and resulted in regular transplantation program since 2009. It currently involves 4 active LuTx centers. Significant improvements in immunosuppressive therapy and more common access to extracorporeal life support (preoperative in a form of bridge to LuTx [3] and intraoperative) resulted in improving the outcomes after LuTx in many centers. Rising median score and higher age at transplantation become more visible in American centers [4, 5], but the prospects of further development of LuTx in Europe are also very promising.

**Scheme 1:**
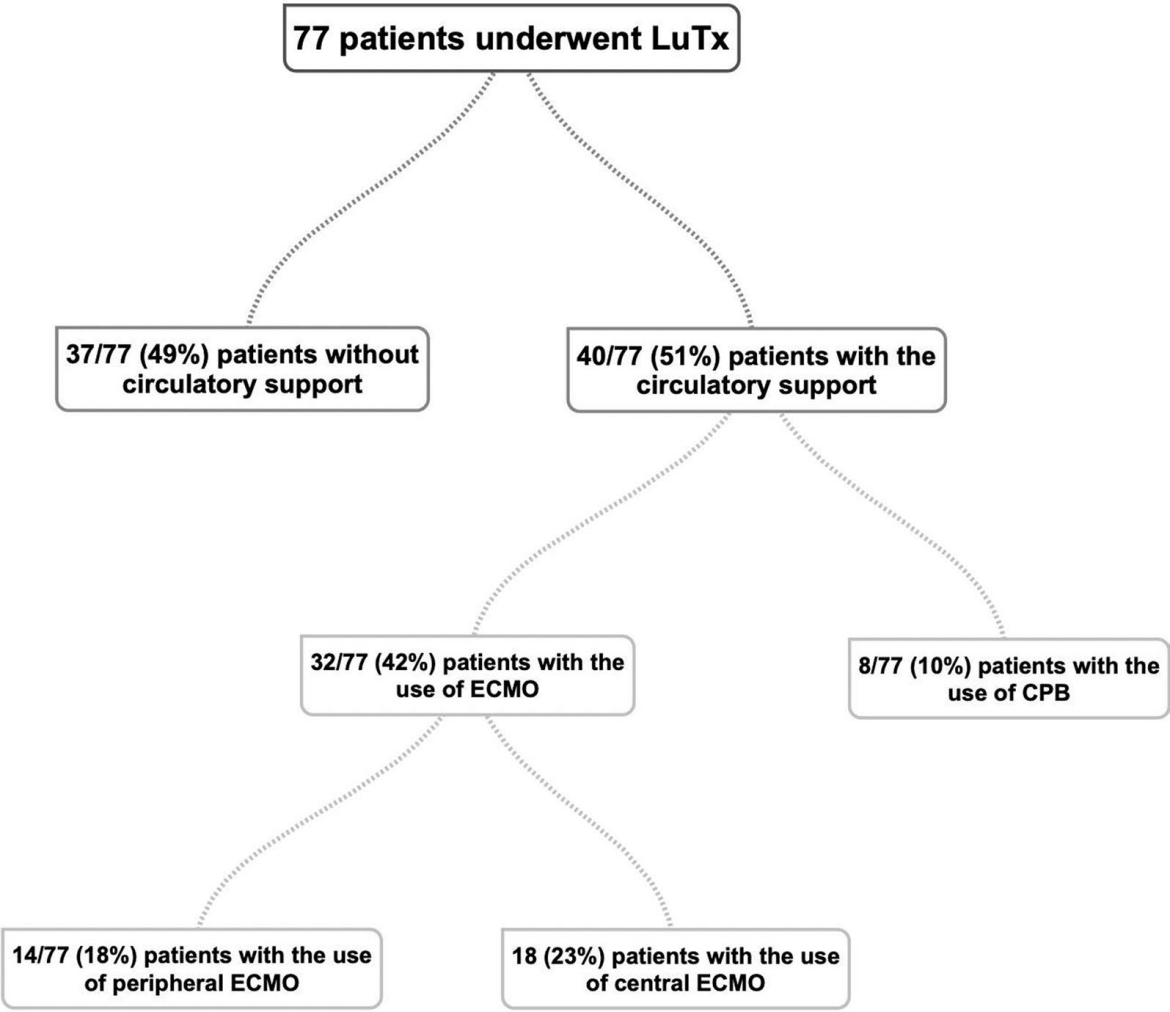
Visual presentation of the analyzed group of patients

Lung transplantation can be performed off-pump, but if the use of extracorporeal circulation is required, extracorporeal membrane oxygenation (ECMO) is more popular for LuTx. Below we analyzed single-center experience of the Department of Thoracic Surgery and Transplantation with circulatory support methods used during operation. In the total group of 77 patients, 40 required the use of extracorporeal assistance methods during the procedure. (see: Scheme 1). Main focus in this study was laid on patients divided in the two sub-groups: 1st with cardiopulmonary bypass (CPB) and 2nd: extracorporeal membrane oxygenation (ECMO)-group according to their use. We analyzed the survival rates and frequency of complications that often appeared afterwards in the form of acute kidney injury (AKI), bleeding, heart arrhythmias or thromboembolic complications.

## Material and methods

### Patients

We reviewed all lung transplantations performed from November 2009 to March 2020. During this 11 years, 77 operations were carried out. 40 patients (52%) required intraoperative extracorporeal assistance. 8 patients (10%) received cardiopulmonary bypass (CPB) and 32 (42%) received extracorporeal membrane oxygenation (ECMO). In the ECMO group, 14 (18%) had peripheral cannulation and 18 (23%) had central one. Mean age, female ratio and basic information about patients’ diseases were listed in Table 1.

**Table 1:** Patient data and indications to lung transplantation

	Off Pump (n = 37)	CPB (n = 8)	Peripheral ECMO (n = 14)	Central ECMO (n = 18)	*p* value
Age ± SD	52 ± 12	37 ± 17	49 ± 12	42 ± 15	0.004
Male n (%)	17 (46%)	6 (75%)	9 (64%)	10 (56%)	0.16
Bilateral lung transplantation n (%)	19 (51%)	6 (75%)	10 (71%)	18 (100%)	0.002
Chronic obstructive pulmonary disease n (%)	17 (46%)	2 (25%)	6 (43%)	0	0.02
Cystic fibrosis n (%)	6 (16%)	3 (36%)	2 (14%)	7 (39%)	0.17
Hypersensivity pneumonitis n (%)	0	1 (13%)	0	2 (11%)	0.14
Usual interstitial pneumonia n (%)	11 (30%)	1 (13%)	3 (21%)	6 (33%)	0.65
Histiocytosis n (%)	0	0	0	1 (6%)	0.52
Idiopathic pulmonary artery hypertension n (%)	0	0	2 (14%)	0	0.27
Retransplantation n (%)	0	1 (13%)	1 (7%)	1 (6%)	0.14
Bronchiechtasis n (%)	1 (3%)	0	0	0	0.48
Alpha1-antitrypsin deficiency n (%)	2 (5%)	0	0	1 (6%)	0.58

*SD* Standard deviation

### Surgical procedures

The standardized protocol was used to evaluate all donor organ quality and function. During bilateral lung transplantation lungs are transplanted sequentially. Pleural cavity was usually opened through anterolateral thoracotomy in the fourth intercostal space. Incision was made from parasternal line to the midaxillary line and depending on single or double LuTx- one or both sides. In some rare cases clamshell technique (transversal sternotomy in both fourth intercostal spaces) was performed instead. Before the procedure perfusion scintigraphy is performed to indicate the organ that is more damaged. If the difference between lungs is 35–65%, it shows that there is essential contrast in perfusion. Consequently, less efficient lung is removed firstly.

The decision to use extracorporeal support was made during operation and it was based on "clamping test". If an attempt of clamping pulmonary artery leads to significant systemic arterial pressure drop, tachycardia, saturation drop or significant increase of pulmonary pressure (measured through right heart catheterization), cardiac support is essential during surgery. After applying ECMO's or CPB cannulas, the first excised lung pulmonary artery branch is clamped and cut, veins are tied and then cut. Suspended main bronchus is cut distally from tracheal carina, pulmonary ligament is transected, and pneumonectomy is completed.

The implantation of donor lung begins with bronchial anastomosis utilizing running absorbable monofilament 4-0 suture, then arterial anastomosis with running 5-0 non-absorbable monofilament suture and finally common venous cuff of donor and recipient with the same running 5-0 non-absorbable monofilament suture are performed.

If necessary, second pneumonectomy and implantation of the graft is performed on the other side. If ECMO or CPB were not used intraoperatively, it is crucial not to clamp the artery of the second excised lung until 45 min elapsed from the reperfusion of the first implanted graft (as a way of protection from pulmonary oedema).

### Extracorporeal support

**Scheme 2:**
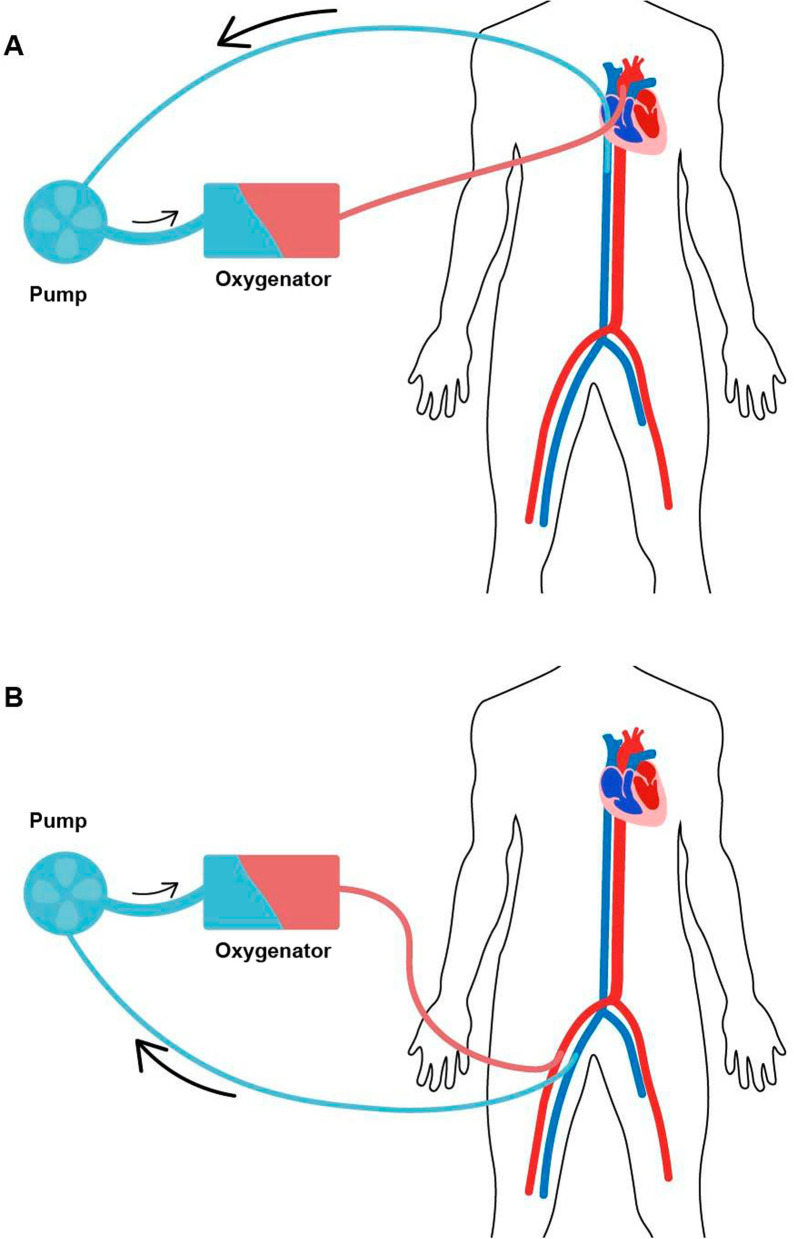
Cannulation of: **a** central ECMO, CPB, **b** peripheral ECMO. **a** Vena cava inferior (through right atrium)- pump- oxygenator- ascending aorta. **b** Femoral vein- pump-oxygenator-femoral artery

Cardiopulmonary bypass and extracorporeal membrane oxygenation were applied using standard procedures (see: Scheme 2).

Due to the high mortality rate of the patients that underwent LuTx with an assistance of CPB, the last operation with cardiopulmonary bypass was in 2016. In 2017 we also discontinued to use peripheral ECMO during surgery and since then- if the use of circulatory support methods during surgery is crucial- we apply only central ECMO.

### Statistical analysis

**Fig. 1:**
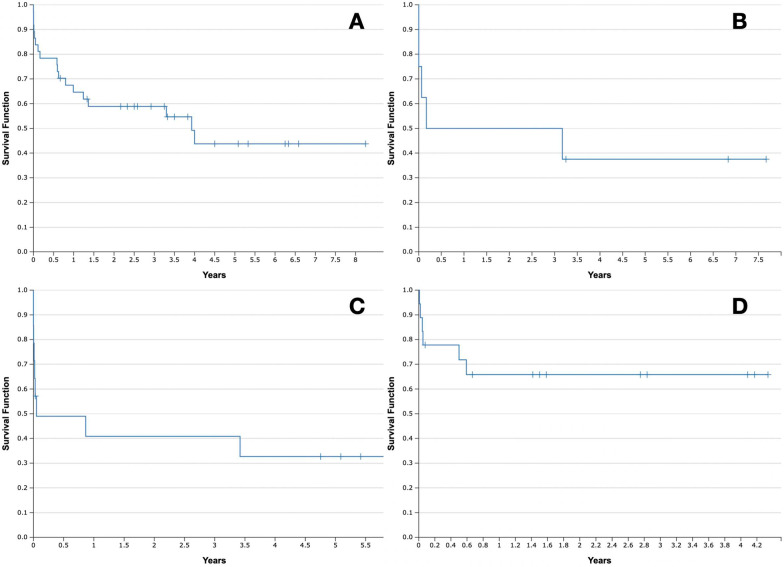
Kaplan–Meier curves. **a** Off pump, **b** CPB, **c** peripheral ECMO, **d** central ECMO

Cumulative survival rates (30 days, 1 year, 3 years, 5 years) of four groups (off pump, CPB, peripheral ECMO, central ECMO) were calculated by the Kaplan–Meier method (Fig. 1). Differences between the groups were evaluated by the Chi- square analysis for discontinuous variables and t-test for continuous variables. *p* values less than 0.05 were considered as statistically significant.

## Results

Patients that underwent LuTx without the extracorporeal life support were statistically older than those who were supported with circulatory devices (*p* = 0.004). In addition, bilateral lung transplantation was performed more often in patients with additional support (*p* = 0.002). Moreover, statistically more patients in the off-pump group suffered from COPD than in groups with the support (*p* = 0.02). The survival rates of study groups were listed in Table 2. We compared postoperative survival rates of four groups: off pump, CPB, peripheral ECMO and central ECMO. The highest 30-days survival was achieved in the group of patients without circulatory support (83%). 1-year, 3-years and 5-years survival rates were 65%, 59% and 44%. In CPB group survival rates were 75%, 50%, 50% and 38% respectively. Accordingly, in peripheral ECMO 50%, 41%, 41% and 33%. In central ECMO group 30-days survival rate was 78%, 1-year, 3-years, 5 years survival was the same (66%). In this group in the time period between 1 and 5 years after the LuTx death has not occurred. The highest perioperative mortality was in CPB and peripheral ECMO groups. Causes of death in short-term postoperative amount of time were: excessive bleeding (4 cases; day 0), heart infraction (1 case; day 1), heart arrythmias with surgical complications of using ECMO (1 case; day 4).

**Table 2:** Survival rates of CPB and ECMO groups

Survival rate	Off pump (%)	CPB (%)	Peripheral ECMO (%)	Central ECMO (%)
30-days	83	75	50	78
1-year	65	50	41	66
3-years	59	50	41	66
5-years	44	38	33	66

## Discussion

The intention of LuTx is to improve the quality of life and to extend the survival of the patients with end-stage respiratory insufficiency. The adequate moment to decide for the lung transplantation is crucial to achieve the success during the procedure [6]. During the lung transplantation, cardiopulmonary reserve of each lung has to be taken into consideration. In the course of the procedure, the patient will be ventilated with just one lung even for several hours. As a result, the remaining lung has to provide enough gas-exchange.

Among common indications for unplanned ECMO, there are hemodynamic instability, impaired gas exchange or right ventricular failure [7]. The main task of extracorporeal life support is to replace heart and lung function. The first attempts on creating such device started between 1935 and 1954 by John Gibbon, Clarence Dennis et al. Throughout time, artificial organs (pump and oxygenator) have been improved to minimize damage of the blood cells. In addition, different strategies in vascular access were invented [8].

CPB or ECMO are being used only in case of patients in serious condition (pulmonary artery hypertension, low cardiopulmonary reserve) who normally would not survive the procedure. Both machines have an essential impact on organism which may cause postoperative complications. To prevent blood clotting, heparin is added and activated clotting time (ACT) is measured to monitor the anticoagulation process. On the contrary, administration of erythrocytes concentrates, plasma and platelet concentrates prevents bleeding complications [9]. In this analysis, statistically more erythrocyte units were applied in patients with the support comparing to off-pump group (*p* = 0.01). Furthermore, more units were used in CPB group comparing to both peripheral and central ECMO (*p* = 0.03).

Cardiopulmonary bypass pumps blood with the use of a roller which might generate any sub pressure but also damages a large number of blood cells and brings risk of air embolism. It requires 200–300 units/kg of heparin with ACT > 400 s [10]. In CPB, blood is drained from the right ventricle and returns to the ascending aorta. This method is similar to the central ECMO, but it is more invasive as a result of rotor pump and higher amount of heparin that is used (Table 3).

**Table 3:** Mean use of erythrocytes units

Group	Mean ± SD	*p* value
Off-pump	8.75 ± 9.09	0.01
CPB	26.14 ± 20.92	
Peripheral-ECMO	18.71 ± 13.53	
Central-ECMO	10 ± 6.93	

*SD* Standard deviation

On the other hand, ECMO uses a centrifugal pump which cannot generate the same sub pressure (400–500 mmHg) [11] as roller in CPB but brings less risks of developing embolism. Compared with CPB, ECMO requires less heparin (50–100 units/kg). There is no difference between the amount of heparin dosage in patients with central or peripheral ECMO. Heparin inhibits the effects of thrombin activity. Therefore, the coagulation process is decreased, so the blood does not form a clot in extracorporeal machines. Furthermore, ECMO set does not have a reservoir which lowers the risks of thromboembolic complications [7].

In central ECMO, blood is drained from the right atrium and vena cava inferior and after flowing through an oxygenator, it returns to ascending aorta [12]. Cannulation process requires sternotomy and is performed in the operating theatre, so access is an advantage of the procedure. The main downside is its invasive character.

In peripheral ECMO, the blood is usually drained from femoral vein and it comes back to the femoral artery. The disadvantages of this method include pumping the blood to the upper part of the body, causing the limb to wither or the Harlequin effect which is a hypoxemia of the upper body parts. Consequently, it might cause cerebral hypoperfusion which makes ECMO therapy unsuccessful [13].

The comparison between ECMO and CPB is shown in Table 4.

**Table 4:** Comparison between ECMO and CPB

	ECMO	CPB
Pump	Centrifugal	Roller
Heparinization	50–100 units/kg	200–300 units/kg
Circuit type	Opened	Closed
Maximal possible time of circulation	Weeks (Veno-venous, as a bridge to LuTx or post LuTx)	Hours
Ischemia of lower limbs	Common (peripheral ECMO)	Rare
Bleeding complications	Less frequent	More frequent
Primary graft dysfunction (PGD)	Less often	More often
Activated clotting time (ACT) necessary	150–170 s	> 400 s
Reservoir	Absent	Present
Open air contact	Absent	Present

*LuTx* Lung transplantation

Veno-venous ECMO is another type of cannulation which is used in respiratory failure but with sufficient cardiac output. Usually blood is drained from femoral vein and reinfused to the jugular internal vein [11]. The patient might be supported with VV ECMO for weeks and the median time of support is increased every year [14]. VV ECMO is not used during lung transplantation, because it supports only lung function and does not support heart function. On the contrary, peripheral VA ECMO might be used in the short term (few days–few weeks).

Moreover, studies based on comparing ECMO and CPB lead to the conclusion that ECMO is related with lower occurrence of primary graft dysfunctions (PGDs), less blood transfusions after LuTx and shorter stay at ICU [15–17]. In our study 40 patients underwent lung transplantation with the intraoperative use of circulatory assistance methods. According to our results (Fig. 1), only patients with central ECMO achieved higher long-term survival rate (5-years) than patients without the use of extracorporeal devices.

Experience of Ius [18] of using extracorporeal support intraoperatively indicates to use ECMO instead of CPB due to its ability to support for weeks. In addition, ECMO is a machine that allows to bridge patients to lung transplantation. In their study, a higher survival rate was achieved in the group of patients that did not require extracorporeal support (4-year survival rate: 73% without support and 69% with support). Moreover, major complications occurred more frequently in the group with the support. Experience with using ECMO and CPB was presented also in the paper written by Aigner [17]. The highest 3-months, 1-year and 3-years survival rates were achieved in the group without support (93.5%; 91.9% and 86.5%). Survival rates for group with intraoperative + prolonged ECMO were 85.4%; 74.2% and 67.6% respectively. The lowest survival rates were achieved in CPB group (74%; 65.9% and 57.7%). According to Weingarten et al. [19] who analyzed several studies related to comparison between outcomes of CPB and ECMO, there is no difference between short-term mortality of these two machines. In case of medium- and long-term mortality, it is increased for CPB. Although there is a shift towards ECMO during lung transplantation, it is still debatable which machine provides better outcomes. No differences were observed in 30-day, 6-months and 1-year mortality between CPB and ECMO groups in the study performed by Bermudez et al. [20]. There has not been a randomized control trial to authorize the dominance of ECMO over CPB.

The use of membrane oxygenator is implemented in the state of hypoxia or hypercapnia, but at the same time, these are the harmful conditions for kidneys. Lung transplantation itself has a large impact on kidneys due to nephrotoxic immunosuppressants administered postoperatively (especially due to use of calcineurin inhibitors) and might be itself a reason for progression of further kidney damaging. In our study, acute kidney injury (AKI) has developed significantly more often in the patients with the use of extracorporeal life support than patients without extracorporeal circulation (*p* = 0.005).

In Zangrillo's study of complications after using ECMO [21], 52% of patients required hemofiltration as a result of renal failure after LuTx. Furthermore, according to Husain-Syed's study [22], AKI appears in more than 70% patients receiving ECMO.

In order to avoid system coagulation, the use of ECMO requires partial heparinization. Higher use of coagulation factors leads to another quite common complication after using ECMO that is related with changes in viscosity and rheological properties of blood. Superposition of the factors like heparin, contact of the blood with air and an inflammatory process caused by flow through cannulas and pump, increases the risk of bleeding, that appears approx. in 33% of cases [21]. It also increases the frequency of thromboembolic complications as a result of platelets damaging and formation of micro embolisms. In our study, complications related to use of heparin due to extracorporeal circulatory assistance methods are: "CPB-patients": 2/8 cases of bleeding (25%), 3/8 cases of thromboembolic complications (37.5%) and in the group of "central ECMO- patients" 8/32 cases of bleeding (25%), 7/32 cases of thromboembolic complications (22%) (see: Table 5).

**Table 5:** Frequency of complications after use of CPB or ECMO during lung transplantation

Complication	Off pump n = 37	CPB n = 8	Central ECMO n = 18	Peripheral ECMO n = 14	*p* value
AKI n (%)	5 (14%)	2 (25%)	8 (44%)	7 (50%)	0.005
Bleeding	6 (16%)	2 (25%)	7 (39%)	1 (7%)	0.36
Thromboembolic complications	2 (5%)	3 (36%)	5 (28%)	2 (14%)	0.02
Post-transplant kidney disease	13 (35%)	2 (25%)	4 (22%)	5 (36%)	0.48
Heart arrhythmia	9 (24%)	1 (13%)	5 (28%)	0	0.32

*AKI* Acute kidney injury

Furthermore, another complications related with the use of extracorporeal membrane oxygenator that might appear according Zangrillo [21] are: bacterial pneumonia (33%), oxygenator replacement according to oxygenator dysfunction (29%), sepsis (26%), liver dysfunction (16%), venous thrombosis (10%), gastrointestinal bleeding (7%) and disseminated intravascular coagulation (5%). In our study, we haven't observed many of these-. there was 1 case of sepsis that caused death on 7th day after LuTx and we observed 3 surgical complications of using ECMO that lead to death in 4th, 7th and 34th day after surgery.

## Limitations

Our study cannot be considered without certain limitations. As this is the retrospective study, the patients were not randomly assigned to the study groups. Especially the CPB group was preoperatively more sick—with more comorbidities, higher pulmonary pressures or after previous thoracic operations. Therefore the results here were worse- it is seen not only in our study, but in the papers of the other LuTx centres as well [23, 24]. The peripheral AV ECMO does not replace the heart function as good as CPB or central ECMO due to Harlequin effect and often has burdened of lower limb ischemia due to arterial cannula insertion.

Also the more experience we gained, the patients were better qualified and sometimes disqualified from the transplantation—all improving the results of our transplanation program.

Till December 2016 we have performed 44 LuTx and from January 2017 till the end of this study period, in summary we have done 77 LuTx. During this study our surgical team remained unchanged. Through all this time we gathered experience, draw conclusions from our results and implemented corrections. We have chosen the best method we experienced—which in our opinion and results is central ECMO. It is also convergent with the Vienna center experience [25, 26].

## Conclusions

The development of extracorporeal circulatory support was one of the significant steps in improving the standard methods in lung transplantation. In this article we present a single center experience based on the 10 years of work and experience by lung transplantation. CPB assistance during LuTx is related with the highest mortality (5-year survival rate 44%) and requires the more units of eryhrocytes comparing to both peripheral and central ECMO (*p* = 0.03). The highest 30-days survival was achieved in the group of patients without circulatory support (83%), but patients with central ECMO achieved the highest long-term survival rate (5-year survival rate 66%- central ECMO vs 44%- off pump). Patients that underwent LuTx required more units of erythrocytes (*p* = 0.01), but less units were needed by patients supported with central ECMO compared to other circulatory support devices (*p* = 0.01). AKI and thromboembolic complications have developed statistically more often in case of patients with the circulatory support (*p* = 0.005, *p* = 0.02 respectively). Frequency of other complications (bleeding, post-transplant kidney disease and heart arrythmia) were comparable (*p* = 0.36, *p* = 0.48, *p* = 0.32 respectively).

According to our results, CPB should be no longer used during LuTx. Among circulatory support methods, central ECMO should be considered to have priority in use during LuTx, although our studies need to be repeated by other groups and with a larger cohort of patients. Also the anticoagulation protocol used during LuTx requires futher development and standarizadion in order to give the best possible short- and long-term survival results and to reduce the amount of complications during and after the surgery.

## Data Availability

Data and materials are available from authors only on reasonable request.

## Abbreviations

LuTx: Lung transplantation
LDLT: Living-donor lung transplantation
SLuTx: Single-lung transplantation
ECMO: Extracorporeal membrane oxygenation
CBP: Cardiopulmonary bypass
AKI: Acute kidney injury
SD: Standard deviation
ACT: Activated clotting time
VV ECMO: Veno-venous ECMO
PGD: Primary graft dysfunction
AV ECMO: Arterio-venous ECMO
